# Eco-Friendly and Biodegradable Biopolymer Chitosan/Y_2_O_3_ Composite Materials in Flexible Organic Thin-Film Transistors

**DOI:** 10.3390/ma10091026

**Published:** 2017-09-03

**Authors:** Bo-Wei Du, Shao-Ying Hu, Ranjodh Singh, Tsung-Tso Tsai, Ching-Chang Lin, Fu-Hsiang Ko

**Affiliations:** Department of Materials Science and Engineering, National Chiao Tung University, 1001 University Road, Hsinchu 30010, Taiwan; dbw6522@gmail.com (B.-W.D.); Gj94ekup8896@gmail.com (S.-Y.H.); chemrjd@gmail.com (R.S.); kenny2870@gmail.com (T.-T.T.)

**Keywords:** non-toxic, organic thin-film transistors (OTFTs), chitosan, Y_2_O_3_, flexible

## Abstract

The waste from semiconductor manufacturing processes causes serious pollution to the environment. In this work, a non-toxic material was developed under room temperature conditions for the fabrication of green electronics. Flexible organic thin-film transistors (OTFTs) on plastic substrates are increasingly in demand due to their high visible transmission and small size for use as displays and wearable devices. This work investigates and analyzes the structured formation of aqueous solutions of the non-toxic and biodegradable biopolymer, chitosan, blended with high-k-value, non-toxic, and biocompatible Y_2_O_3_ nanoparticles. Chitosan thin films blended with Y_2_O_3_ nanoparticles were adopted as the gate dielectric thin film in OTFTs, and an improvement in the dielectric properties and pinholes was observed. Meanwhile, the on/off current ratio was increased by 100 times, and a low leakage current was observed. In general, the blended chitosan/Y_2_O_3_ thin films used as the gate dielectric of OTFTs are non-toxic, environmentally friendly, and operate at low voltages. These OTFTs can be used on surfaces with different curvature radii because of their flexibility.

## 1. Introduction

For two decades, the global developments of the electronics industry have focused on flexible electronic devices, such as curved full-color displays [1,2] integrated sensors [3], flexible solar cells, and the amazing achievement of E-paper [4,5]. Using a solution-based process achieves many advantages that are cost-effective and simple to fabricate, and produces mechanically flexible thin-film transistors compared to conventional semiconductor technologies, which depend on vacuum-based thin film fabrication [6,7]. In the past decade, eco-friendly, biocompatible, and green materials have been the subject of many economic and scientific projects [8,9,10], and have caused less damage to the environment. Oxide thin films under low annealing temperatures have been fabricated by using an inexpensive “water-inducement” technique [11] that combines a high-k-value YO_X_ dielectric material with an eco-friendly water-inducement process [12]. Because of their superior performance, organic thin-film transistors (OTFTs) can be used to replace conventional thin-film transistors (TFTs). Chitin is a natural amino polysaccharide and is the largest nitrogenous natural organic compound on the planet after protein and the cellulose polysaccharides found in nature [13]. It has many outstanding characteristics, such as biocompatibility, non-toxicity, biodegradability, antimicrobial activity, and excellent mechanical strength, which make it suitable for use in the biomedical field [14,15]. Chitosan can be transformed into chitin and has the same excellent characteristics, such as biocompatibility, biodegradability, non-toxicity, antimicrobial activity, and an outstanding film-forming ability [16,17,18]. Therefore, in previous studies, chitosan has also been used as dielectric layer in organic transistors [19,20]. Yttrium (III) oxide (Y_2_O_3_) is a type of rare earth oxide that is non-toxic, thermodynamically stabile, stabile at high temperature (T_m_ = 2430 °C), and has a high dielectric constant (ε = 15~18), light transparency, and a linear transmittance in the infrared spectra. Y_2_O_3_ is commonly known as a high-k dielectric material that can replace SiO_2_, because it has a high-k dielectrics value and a phase with cubic symmetry. Its lattice constant, a = 10.6 Å, is two times as large as the lattice constant of Si (a = 5.43 Å). Y_2_O_3_ can be deposited by different deposition techniques, including pulsed laser deposition (PLD) [21], sputtering metal-organic chemical vapor deposition (MOCVD) [22], and electron beam evaporation [23].

Therefore, due to its high-k-value, non-toxicity, and biocompatibility, Y_2_O_3_ nanoparticles were blended into the chitosan solution in this study to improve their dielectric properties and pinholes. To achieve good electrical performance with the chitosan-based metal-insulator-metal (MIM) structure, various concentrations of Y_2_O_3_ nanoparticles were blended into the chitosan to decrease the leakage current and improve the depth of the pinholes. Chitosan thin films have an electric-double-layer effect that gives OTFTs the property of low-voltage operation. Furthermore, the thin films of chitosan blended with Y_2_O_3_ nanoparticles were used as the dielectric material in OTFTs, and the performance of these OTFTs was enhanced.

## 2. Experimental 

Yttrium (III) oxide (Y_2_O_3_) was provided by Alfa-Aesar (Heysham, UK). Chitosan, poly(3-hexylthiophene) (P3HT) and acetic acid were provided by Sigma-Aldrich (St. Louis, MO, USA). All other reagents and anhydrous solvents were obtained from local suppliers and used without further purification, unless otherwise noted.

**Figure 1:**
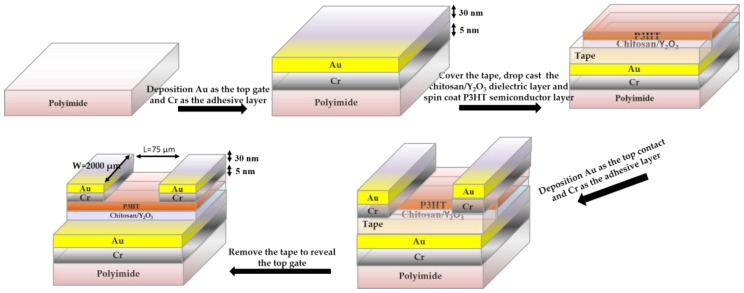
The fabrication process of the bottom-gate top-contact flexible organic thin-film transistor.

The Y_2_O_3_/chitosan thin film as the dielectric gate of the flexible OTFT, and P-type organic semiconductor, poly(3-hexylthiophene) (P3HT), as the semiconductor layer on polyimide substrate were demonstrated in this study. The basic process flow for the fabrication of this flexible device is shown in Figure 1. Moreover, the performance of the flexible OTFT during bending tests with different curvature radii was also observed. In detail, a 5-nm Cr metal layer was deposited as an adhesive layer on the polyimide film by thermal deposition and then a 30-nm Au layer was deposited on the adhesive layer as the bottom gate. The adhesive layer was used to stabilize the Au layer and guarantee that the device would be stable under bending tests. The polyimide substrate was first cleaned before placing it into thermal coater chamber, after which a Cr and Au metal layer was deposited. Before the dielectric layer was deposited, we covered the adhesive tape on the bottom Au electrode, for the bottom gate can only be exposed in the final step.

Chitosan (deacetylated ≥75%) was dissolved in aqueous acetic acid (0.5 wt %) and heated with a hot plate at 50 °C for 24 h, following which its solutions of various concentrations were filtered with a 25-mm syringe filter containing a 0.45-μm polyvinylidene difluoride (PVDF) membrane. The Y_2_O_3_ nanoparticles were blended with deionized water at a ratio of 0.5 wt % and the hybrid solution was obtained by mixing chitosan aqueous solution with Y_2_O_3_ nanoparticles aqueous solution, with a specific volume ratio CS:Y_2_O_3_ (20:1). In other words, the weight percentage of Y_2_O_3_ in the blended solutions was 0.023 wt %. The drop casting was used to form the 0.023 wt % Y_2_O_3_/chitosan film on the bottom-gate as the dielectric gate, which was then dried in an oven at room temperature for 24 h.

After depositing the dielectric gate, a P3HT channel layer was deposited on the Y_2_O_3_/chitosan dielectric film by spin-coating at 1200 rpm for 30 s and then 1500 rpm for 30 s. The polyimide substrate was placed in an oven at 60 °C to remove residual solvent in the P3HT active channel layer. Finally, a 5-nm Cr and a 30-nm Au layer were deposited with a mask to form the source and drain top contacts. Cr metal was used as an adhesive layer between the P3HT channel layer and Au contacts as before. In the meantime, the bottom Au electrode was revealed by carefully removing the tape. Therefore, as the above procedure was finished, the bottom-gate top-contact flexible organic thin-film transistor was successfully designed.

## 3. Results and Discussion

### 3.1. Materials and Films Characterization

**Figure 2:**
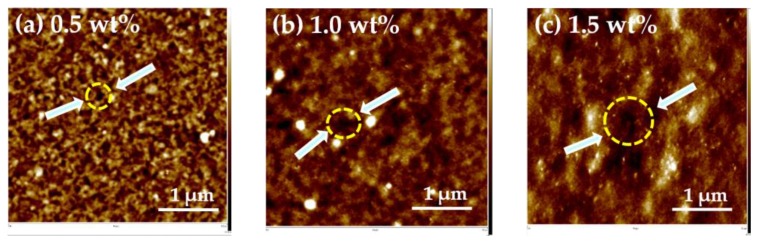
(**a**) 0.5 wt % chitosan shows many small holes (**b**) 1.0 wt % chitosan shows a few middle-sized holes and (**c**) 1.5 wt % chitosan shows many large holes.

Chitosan (deacetylated ≥75%) was dissolved in aqueous acetic acid (0.5 wt %) and heated by using a hot plate at 55 °C for 24 h. The impurities in the chitosan solutions of various concentrations were filtered using a 25-mm syringe filter with a 0.45-µm PVDF membrane. The chitosan solution with various concentrations was transferred by spin-coating onto separate single silicon substrates that were already coated with aluminum metal as an electrode by spin-coating. Then, we removed the water in the chitosan thin film by heating on a hot plate at 80 °C for 1 h. Finally, we deposited the aluminum metal as the top electrode on the chitosan, which formed a so-called MIM structure. We found that the lower leakage current was 6.827 × 10^−10^ A at an applied voltage of 2 V in the MIM based on a 1.0 wt % chitosan thin film, and observed that the 1.0 wt % chitosan film had the lowest leakage current. This result was attributed to the size and the number of the pinholes in the surface of the chitosan, as shown in Figure 2. The thin film with 0.5 wt % chitosan had many small pinholes (10–20 nm), as shown in Figure 2a, and the thin film with 1.5 wt % chitosan had some large pinholes (80–100 nm), as shown in Figure 2c. The thin film with 1.0 wt % chitosan had the optimized hole size (30–50 nm) and number of holes, as shown in Figure 2b; this is why the 1.0 wt % sample had the lowest leakage current.

**Figure 3:**
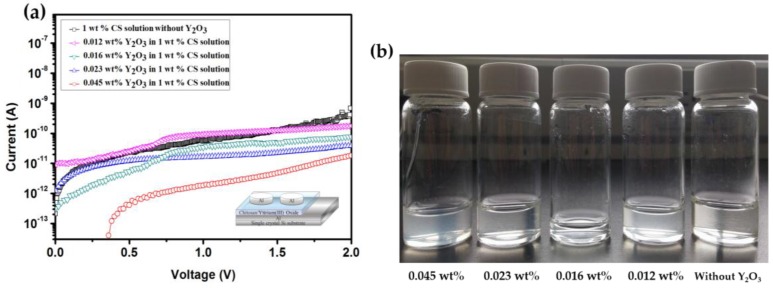
(**a**) Leakage current of the thin films measured with various concentrations of Y_2_O_3_ solutions (without Y_2_O_3_, 0.012 wt %, 0.016 wt %, 0.023 wt %, 0.045 wt %) in 1 wt % chitosan solution (**b**) Pictures showing the various weight percentages of Y_2_O_3_ in the mixed solution.

**Figure 4:**
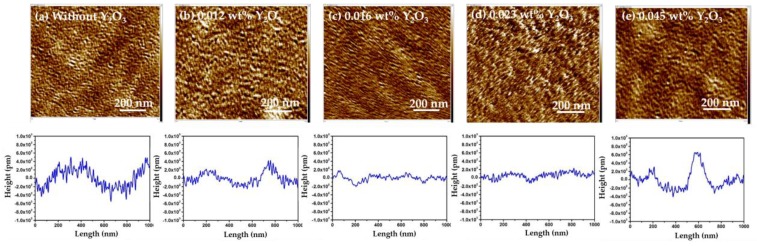
AFM morphology and profile depth of the pinholes in the surface of the Y_2_O_3_/chitosan thin film with various Y_2_O_3_ concentrations: (**a**) Without Y_2_O_3_; (**b**) 0.012 wt %; (**c**) 0.016 wt %; (**d**) 0.023 wt %; and (**e**) 0.045 wt %.

The high-k-value Y_2_O_3_ nanoparticles were blended into a 1 wt % chitosan water solution with various weight percentages of Y_2_O_3_ (from 0.012 wt % to 0.016 wt %, 0.023 wt % and 0.045 wt %). While the weight percentage increased, the leakage current decreased. The blended 0.045 wt % Y_2_O_3_ in 1 wt % chitosan thin film had the lowest leakage current of 1.81 × 10^−11^ A, as shown in Figure 3a. The pure chitosan thin films were almost transparent, the weight percentage of the Y_2_O_3_ increased, as shown in Figure 3b. In the meantime, we discovered a decrease in the relative depth (from 1.025 to 0.356 nm) when the concentration of the Y_2_O_3_ increased, as shown in Figure 4a–d. However, the relative depth and roughness were increased when the weight percentage of Y_2_O_3_ reached 0.045 wt %, as shown in Figure 4e. The purpose of blending the Y_2_O_3_ nanoparticles into the chitosan thin film was not to only reduce the leakage current, but also to improve the pinholes at the surface. 

**Figure 5:**
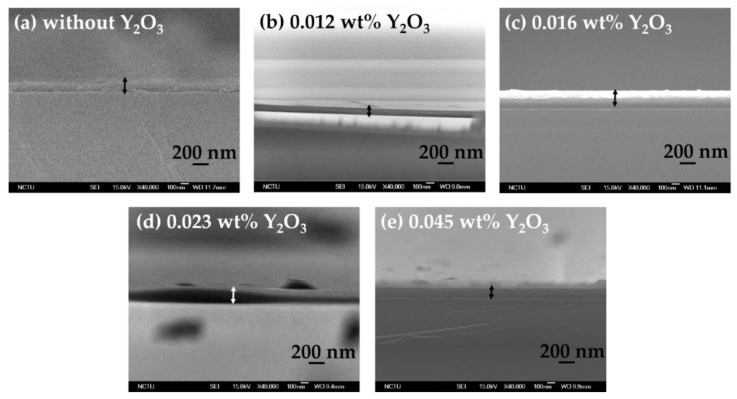
Cross-section images of the blended Y_2_O_3_/chitosan thin films: (**a**) Without Y_2_O_3_; (**b**) 0.012 wt %; (**c**) 0.016 wt %; (**d**) 0.023 wt %; and (**e**) 0.045 wt %.

**Figure 6:**
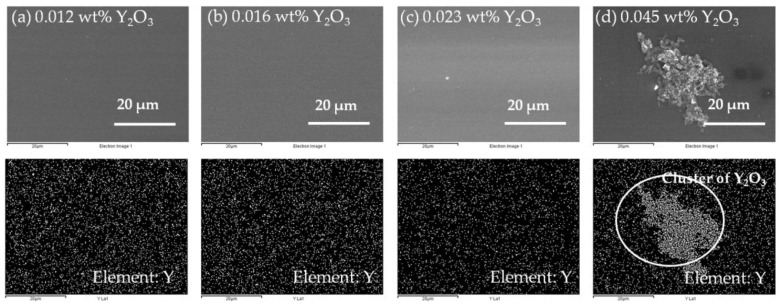
EDX images showing the distribution of yttrium: (**a**) 0.012 wt %; (**b**) 0.016 wt %; (**c**) 0.023 wt %; and (**d**) 0.045 wt %.

The cross-sectional images of the blended Y_2_O_3_/chitosan thin films are shown in Figure 5, and the thicknesses of the blended thin films were approximately 120 nm–200 nm. We also analyzed the distribution of the Y_2_O_3_ nanoparticles in the surface of the blended thin films by energy-dispersive X-ray spectroscopy (EDX, JEOL, Freising, Germany). We discovered that the 0.012 wt %, 0.016 wt %, and 0.023 wt % blended thin films showed a uniform dispersion of the Y_2_O_3_ nanoparticles, as shown in Figure 6a–c. The Y_2_O_3_ nanoparticles attracted each other and clusters formed when the weight percentage of Y_2_O_3_ reached 0.045 wt % (Figure 6d). In Figure 4e, we measured the relatively large profile depth of 1.051 nm, and we attributed this phenomenon to the clustering of the Y_2_O_3_ nanoparticles. It was observed that the 0.023 wt % thin film had smoothest surface, and its pinholes were much improved. 

**Figure 7:**
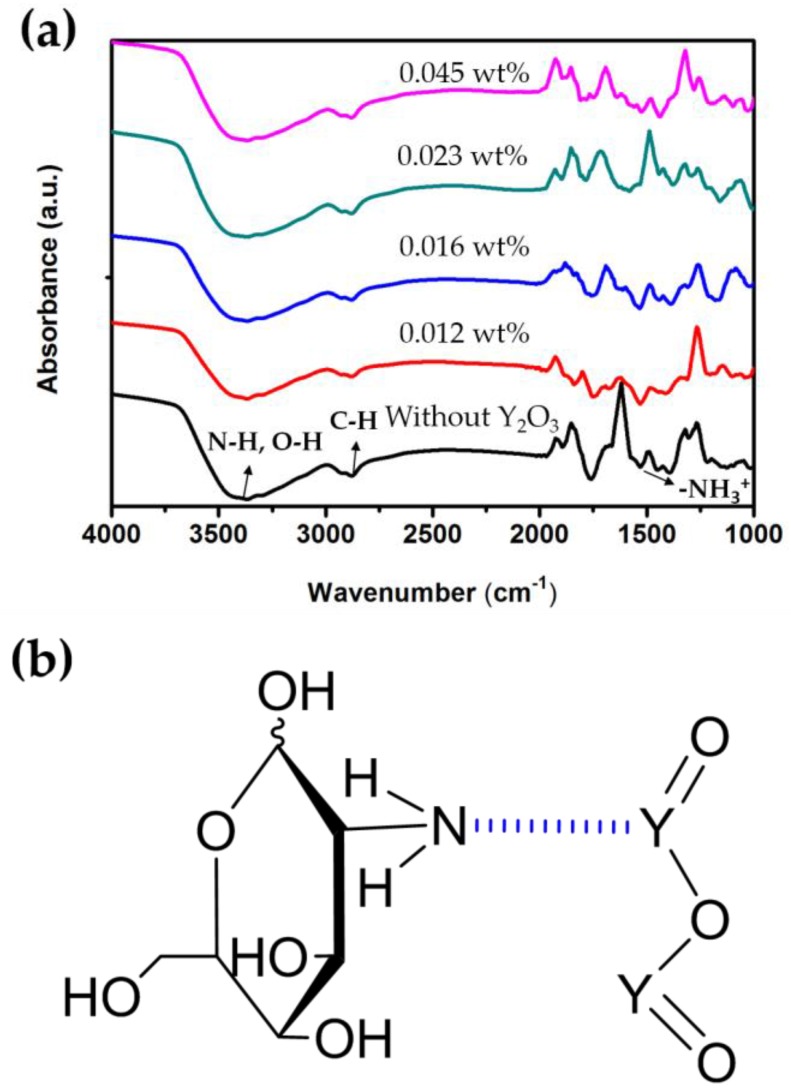
(**a**) FTIR spectra of the blended Y_2_O_3_/chitosan thin films; (**b**) Schematic diagram of hydrogen bonding between the Y_2_O_3_ nanoparticles and the amine group of chitosan.

The FTIR spectra analysis (PerkinElmer, Waltham, MA, USA) of the blended Y_2_O_3_/chitosan thin films was used to obtain information on the chemical bonding, as shown in Figure 7a. The pure chitosan thin film showed broad absorption peaks at 3000–5000 cm^−1^ that were attributed to NH_2_ asymmetric stretching and the hydrogen-bonded OH. The peak at approximately 2879 cm^−1^ was attributed to the CH_3_ asymmetric stretching vibrations, and the absorption peak at 1541 cm^−1^ was attributed to the asymmetric bending modes [24,25,26] of NH_3_^+^. Figure 7a also shows the FTIR spectra analysis of all the blended Y_2_O_3_/chitosan thin films, which are listed in Table 1. The NH_3_^+^ was assigned to the bending frequency at 1541 cm^−1^ for pure chitosan, which shifted to a higher frequency at 1559 cm^−1^ and 1580 cm^−1^ for Y_2_O_3_ = 0.012 wt % and 0.016 wt %, respectively, then shifted to a higher frequency at 1598 cm^−1^ for Y_2_O_3_ = 0.023 wt % and shifted to a lower frequency as 1578 cm^−1^ for Y_2_O_3_ = 0.045 wt %. The shifts were due to the H-bonds between the oxygen of yttrium (III) oxide and the amine of chitosan, as shown in Figure 7b. The AFM morphology (Veeco, Plainview, NY, USA) also proved that a dense structure and fewer pinholes were formed by blending Y_2_O_3_ nanoparticles into the thin film, as shown in Figure 4.

### 3.2. Electric Characteristics of the Flexible Organic Thin Film Transistor

**Figure 8:**
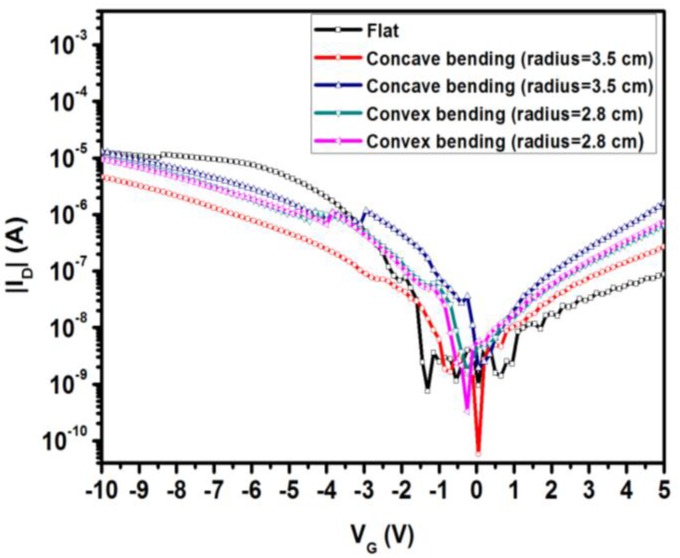
Comparison of the electrical characterization of the P3HT-based organic thin-film transistors (OTFTs) with a 0.023 wt % blended Y_2_O_3_/chitosan dielectric gate for bending tests at different bending radii and without bending.

We investigated the electrical properties of the flexible P3HT-based OTFTs with a 0.023 wt % blended Y_2_O_3_/chitosan dielectric gate on the polyimide substrate. Figure 8 shows the transfer plots for concave and convex bending of the OTFTs with concave bending radii, R, of 3.5 cm and 2.8 cm and convex bending radii, R, of 3.5 cm and 2.8 cm. In this figure, the electrical characterization of the OTFTs on polyimide substrates without bending is similar to that of the OTFTs on silicon wafers. After concave bending, the I_off_ value decreased from 7.421 × 10^−10^ A to 5.740 × 10^−11^ A due to extrusion, causing a decrease in the size of the pinholes. On the other hand, after convex bending, the I_off_ value increased to 1.722 × 10^−9^ A. No matter the direction of bending, the I_on_ value would decrease. In Figure 8, we compared the electrical characterization of the OTFTs with different bending radii (3.5 cm and 2.8 cm), and the comparison chart is listed in Table 2. The output characterization (I_DS_-V_DS_) of this device, as shown in Figure S1.

## 4. Conclusions

A solution-based processed and low-voltage operating P3HT-based OTFT with a Y_2_O_3_/chitosan gate dielectric layer was demonstrated in this study. To improve the electrical performance of the chitosan-based MIM, various concentrations of Y_2_O_3_ nanoparticles were blended into the chitosan, which achieved a decreased leakage current and improved the depth of the pinholes. Furthermore, the P3HT-based OTFT with a 0.023 wt % blended Y_2_O_3_/chitosan gate dielectric layer was manufactured on polyimide for bending tests. The electrical performance of the flexible device incurred no obvious changes except for a slight increase in the leakage current and off current. The non-toxic and eco-friendly biopolymer chitosan, blended with Y_2_O_3_ nanoparticles, was successfully used in flexible OTFTs as the gate dielectric, enabling the OTFT to operate under low voltages, and producing a I_on_/I_off_ ratio of 10^5^ at a gate voltage of −10 V and a drain voltage of −1 V. 

## Figures and Tables

**Table 1:** FTIR spectra analysis of the blended Y_2_O_3_/chitosan thin film.

Y_2_O_3_ wt %	Wave Number (cm^−^^1^)	CH Stretching	NH_3_^+^ Bending
	O–H and N–H Stretching Broad Absorption Peaks		
n/a	3368	2879	1541
0.012	3368	2880	1559
0.016	3368	2880	1580
0.023	3367	2880	1598
0.045	3367	2879	1578

**Table 2:** Electrical characterization of the P3HT-based OTFTs with a 0.023 wt % blended Y_2_O_3_/chitosan dielectric gate on the polyimide substrate for bending tests.

Condition	V_th_ (V)	I_on_ (A)	I_off_ (A)	I_off_/I_on_ Ratio	Mobility (cm^2^/Vs)
Flat	−2.0	$1.268\times 10^{-5}$	$7.421\times 10^{-10}$	$10^{5}$	$2.50\times 10^{-2}$
Concave bending 3.50 (cm)	−2.5	$4.592\times 10^{-6}$	$5.740\times 10^{-11}$	$10^{5}$	$3.33\times 10^{-2}$
Concave bending 2.85 (cm)	−2.7	$1.298\times 10^{-5}$	$1.913\times 10^{-9}$	$10^{4}$	$2.70\times 10^{-2}$
Flat	−2.1	$1.294\times 10^{-5}$	$4.571\times 10^{-10}$	$10^{5}$	$1.87\times 10^{-4}$
Convex bending 3.50 (cm)	−3.0	$9.899\times 10^{-6}$	$1.722\times 10^{-9}$	$10^{3}$	$8.53\times 10^{-2}$
Convex bending 2.85 (cm)	−3.3	$9.480\times 10^{-6}$	$3.296\times 10^{-10}$	$10^{3}$	$4.80\times 10^{-2}$
Flat	−1.5	$1.059\times 10^{-5}$	$2.539\times 10^{-9}$	$10^{4}$	$3.33\times 10^{-2}$

## Supplementary Materials

The following are available online at www.mdpi.com/1996-1944/10/9/1026/s1. Figure S1: Output characterization of P3HT-based OTFTs with 0.023 wt % blended Y_2_O_3_/chitosan dielectric gate.
