# Supplementary material for: Eco-Friendly and Biodegradable Biopolymer Chitosan/Y2O3 Composite Materials in Flexible Organic Thin-Film Transistors
Source: Materials (Basel). 2017 Sep 3;10(9):1026. doi: 10.3390/ma10091026 (PMC5615681; doi:10.3390/ma10091026)
Supplement: Supplementary file 1 [file materials-10-01026-s001.docx]

**Supporting Information**

Article

Eco-Friendly and Biodegradable Biopolymer Chitosan/Y_2_O_3_ Composite Materials in Flexible Organic Thin-Film Transistors

Bo-Wei Du, Shao-Ying Hu, Ranjodh Singh, Tsung-Tso Tsai, Ching-Chang Lin * and
Fu-Hsiang Ko *

Department of Materials Science and Engineering, National Chiao Tung University, 1001 University Road, Hsinchu 30010, Taiwan; dbw6522@gmail.com (B.-W.D.); Gj94ekup8896@gmail.com (S.-Y.H.); chemrjd@gmail.com (R.S.); kenny2870@gmail.com (T.-T.T.)

***** Correspondence: kyo@nctu.edu.tw (C.-C.L.); fhko@mail.nctu.edu.tw (F.-H.K.);
Tel.: +886-920-285-453 (C.-C.L.); +886-910-399-192 (F.-H.K.)

The following picture shows output characteristic (I_DS_-V_DS_) of flexible P3HT-based OTFTs blended 0.023 wt% Y_2_O_3_/chitosan as the dielectric layer, with V_GS_ varied from -1 to -7 V, in steps of -2 V. The I_DS_ is $-3.1213\times{10}^{-9}$ and $-3.2653\times{10}^{-9}$ A for V_GS_ is -1 and -3 V, and I_DS_ is $-3.8484\times{10}^{-9}$ and $-4.6808\times{10}^{-9}$ A for V_GS_ is -5 and -7 V, respectively.


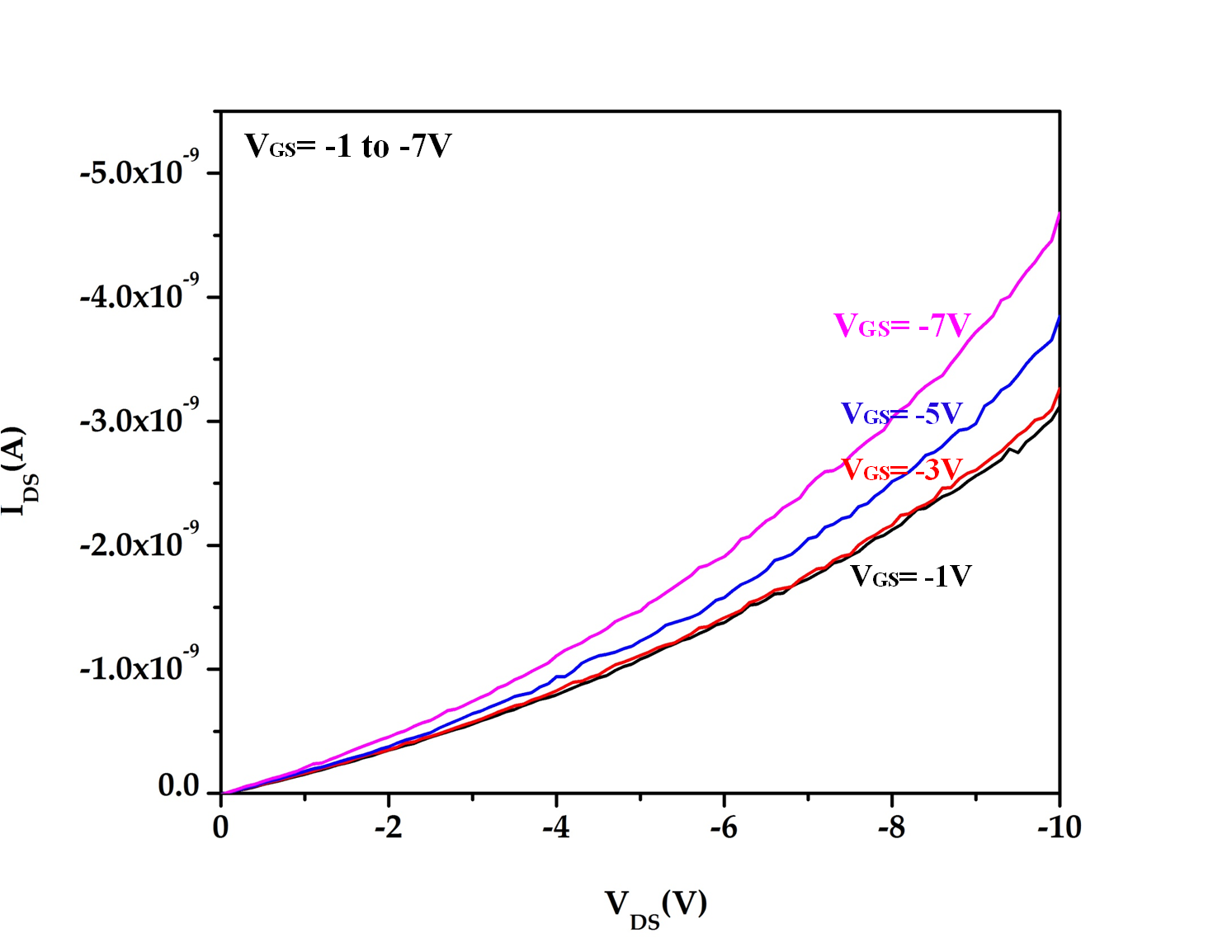


Figure S1. Output characterization of P3HT-based OTFTs with 0.023 wt% blended Y_2_O_3_/chitosan dielectric gate.
